# A Network Study of Chinese Medicine Xuesaitong Injection to Elucidate a Complex Mode of Action with Multicompound, Multitarget, and Multipathway

**DOI:** 10.1155/2013/652373

**Published:** 2013-08-24

**Authors:** Linli Wang, Zheng Li, Xiaoping Zhao, Wei Liu, Yufeng Liu, Jihong Yang, Xiang Li, Xiaohui Fan, Yiyu Cheng

**Affiliations:** ^1^Pharmaceutical Informatics Institute, College of Pharmaceutical Sciences, Zhejiang University, Hangzhou 310058, China; ^2^State Key Laboratory of Modern Chinese Medicine, Tianjin University of Traditional Chinese Medicine, Tianjin 300193, China; ^3^College of Preclinical Medicine, Zhejiang Chinese Medical University, Hangzhou 310053, China

## Abstract

Chinese medicine has evolved from thousands of years of empirical applications and experiences of combating diseases. It has become widely recognized that the Chinese medicine acts through complex mechanisms featured as multicompound, multitarget and multipathway. However, there is still a lack of systematic experimental studies to elucidate the mechanisms of Chinese medicine. In this study, the differentially expressed genes (DEGs) were identified from myocardial infarction rat model treated with Xuesaitong Injection (XST), a Chinese medicine consisting of the total saponins from *Panax notoginseng* (Burk.) F. H. Chen (Chinese Sanqi). A network-based approach was developed to combine DEGs related to cardiovascular diseases (CVD) with lines of evidence from the literature mining to investigate the mechanism of action (MOA) of XST on antimyocardial infarction. A compound-target-pathway network of XST was constructed by connecting compounds to DEGs validated with literature lines of evidence and the pathways that are functionally enriched. Seventy potential targets of XST were identified in this study, of which 32 were experimentally validated either by our *in vitro* assays or by CVD-related literatures. This study provided for the first time a network view on the complex MOA of antimyocardial infarction through multiple targets and pathways.

## 1. Introduction

Chinese medicine has evolved from thousands of years of empirical applications and experiences of combating diseases. It has been profoundly influencing the life and healthcare of the Chinese throughout the history [1, 2]. Chinese medicine is becoming more widely used for preventing and curing the disease clinically and improving healthcare nutritionally [3, 4]. Much progress has been made in better understanding the chemical constituents of Chinese medicine and their therapeutic mechanisms during the last few decades [5]. Chemical constituents, especially the major constituents of some Chinese medicines, have been separated and identified. Chinese medicine is usually a multicomponent system whether it is a herb or a formulae consisting of several types of medicinal herbs or minerals [3, 6, 7]. The therapeutic effects of Chinese medicine rely mostly on the composition and content of the effective constituents [8]. But the effect and functional mechanism of these effective substances in the therapy of Chinese medicine is oftentimes unclear or not fully understood. 

It has become more recognized that Chinese medicine produces the healing efficacy in a more holistic way [9–11]. However, researchers typically focus on the mechanism of either the whole formulae or a few representative components (not necessarily effective ingredients) in single pathological model or mechanism. It is difficult to study the concrete mechanism of a whole formula as it is a mixture, while in the latter case the highly dynamic interaction between ingredients is missing. A few existing studies of multitarget property of Chinese medicine are limited to either computational predictions [12] or mechanism of the whole formulae [13]. 

With the finishing of human genome sequencing and development of various omics technologies, genome wide profiling has enabled systems-level investigation of the mechanisms of actions (MOA) of Chinese medicine [8, 14]. Network pharmacology aims at understanding the effect of drugs in biological system in a holistic manner providing new perspectives in understanding the complex interactions between drug components and biological molecules [15–17]. A combination of these methods may open up new avenues for uncovering the molecular mechanisms underlying the therapeutic efficacy of Chinese medicine in the context of biological networks [18]. 

Xuesaitong Injection (XST) is one of the best selling prescription of Chinese medicine in China [19]. It is a preparation consisting of the total saponins extracted from *Panax notoginseng* (Burk.) F. H. Chen (Chinese Sanqi). There is a wide range of clinical efficacy of XST being extensively used for the treatment of cardiocerebrovascular diseases such as myocardial infarction, cerebral infarction, thrombosis, and coronary heart disease in China [20–27]. Notoginsenoside R1, ginsenoside Rg1, ginsenoside Rb1, ginsenoside Rd, and ginsenoside Re have been found to be the major effective ingredients in our previous study. However their potential targets and the molecular regulatory mechanisms remain to be systematically elucidated. 

**Figure 1:**
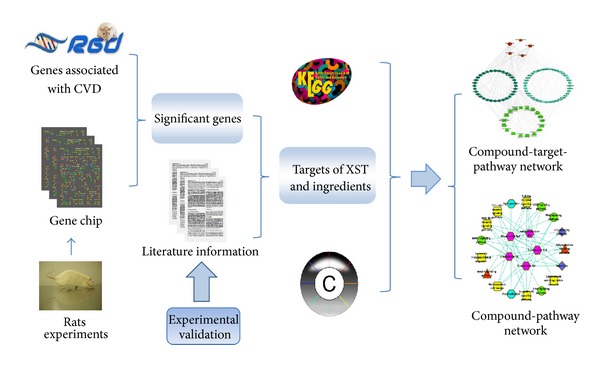
A framework of network pharmacology study of Chinese medicine. The genes involved in anti-MI mechanism of XST were first detected with microarray gene expression analysis and rat genome database (RGD). The target information of the major ingredients was collected from literatures manually and validated experimentally *in vitro*. The compound-target-pathway network and compound-pathway network on anti-MI of XST were constructed to illustrate the multicompound, multitarget, multipathway, and multi-MOA regulatory mechanism of XST.

In this study, we developed a network-based method combining differential gene expression analysis and confirmed literature lines of evidence to study the multicomponent, multitarget, multipathway, and multi-MOA mechanism of XST on antimyocardial infarction (MI). As shown in Figure 1, the genes involved in anti-MI mechanism of XST were first detected with microarray gene expression analysis. Their associations with cardiovascular diseases (CVDs) were evaluated based upon information in rat genome database (RGD) [28]. Considering the amount of studies on major ingredients of XST, we also collected the target information of the five major ingredients in literatures manually. If a CVD associated and differentially expressed gene is also found to be influenced directly by a major ingredient of XST in the literature, it is then considered as a potential target of the compound and XST in this study. Furthermore, some of the potential targets were also experimentally validated. Finally we constructed the compound-target-pathway network on anti-MI of XST to illustrate its multicompound, multitarget, multipathway, and multi-MOA regulatory mechanism. 

## 2. Materials and Methods

### 2.1. Chemicals and Reagents

Chloral hydrate was purchased from Tianjin Kemiou Chemical Reagent Co. (Tianjin, China). The XST lyophilized powder, one of the major types of XST Injection in clinical practices (Batch no. s120425-1), was manufactured by Heilongjiang Zhenbaodao pharmaceutical Co. Ltd. (Heilongjiang, China). Ginsenoside Rg1 (Batch no. W13-5-1) was purchased from Zhongxin Innova Laboratories (Tianjin, China). Ginsenoside Rb1 (Batch no. 120420), notoginsenoside R1 (Batch no. 120325), ginsenoside Rd (Batch no. 120507), and ginsenoside Re (Batch no. 120510) were purchased from Ronghe Pharmaceutical Technology Development Co. Ltd. (Shanghai, China). Lipopolysaccharide (LPS) and dimethyl sulfoxide (DMSO) were purchased from Sigma Chemical Co. (St. Louis, MO, USA). The primary antibody for iNOS was obtained from Abcam (Cambridge, UK), *β*-actin was obtained from Beyotime Institute of Biotechnology (Jiangsu, China), and all the secondary antibodies were obtained from Shuji biotechnology (Shanghai, China). Penicillin and streptomycin were purchased from BIO BASIC INC. (Shanghai, China). Dulbecco's Modified Eagle's Medium (DMEM), fetal bovine serum (FBS), and 0.25% trypsin-EDTA for the cell culture were purchased from GIBCO (USA). RIPA lysate and PMSF were obtained from Beyotime Institute of Biotechnology (Jiangsu, China). Protease inhibitor cocktail tablets and phosphatase inhibitor cocktail tablets were purchased from Roche Diagnostic GmbH (Mannheim, Germany).

### 2.2. Genome-Wide Transcriptomic Experiment

#### 2.2.1. Myocardial Infarction Rat Model

Male Sprague-Dawley rats (230–295 g) used in this experiment were purchased from Weitong-Lihua Experimental Animal Co. Ltd. (Beijing, China). Myocardial infarction was produced by occlusion of the left anterior descending coronary artery. Rats were randomly assigned to three groups: control group (the ligation suture was placed in the heart, but without ligation), myocardial infarction group (MI), and XST treatment group (MI + XST). 5% ethanol-saline solutions (v/v) of XST (150 mg/kg body wt) were given to XST treatment group by intravenous injection once daily and consecutively for 7 days, respectively. 5% ethanol-saline solutions (v/v) were given to the control group and MI group. The administration procedure for rats in this study is in accordance with clinical use. On the eighth day, rats were anaesthetized by intraperitoneal injection of chloral hydrate (12%, 360 mg/kg body wt). Then the risk region in rat heart was collected and stored in liquid nitrogen.

#### 2.2.2. Microarray Experiment

Total RNA was extracted using TRIZOL Reagent (Life technologies, Carlsbad, CA, USA) following the manufacturer's instructions and amplified, labeled, and purified using GeneChip 3′IVT Express Kit (Affymetrix, Santa Clara, CA, USA) following manufacturer's instructions to obtain biotin labeled cRNA. Array hybridization and wash were performed using GeneChip Hybridization, Wash, and Stain Kit (Affymetrix, Santa Clara, CA, USA) in Hybridization Oven 645 (Affymetrix, Santa Clara, CA, USA) and Fluidics Station 450 (Affymetrix, Santa Clara, CA, USA) following manufacturer's instructions. Slides were scanned by GeneChip Scanner 3000 (Affymetrix, Santa Clara, CA, USA) and Command Console Software 3.1 (Affymetrix, Santa Clara, CA, USA) with default settings. Raw data were stored in ArrayTrack 3.5.0 [29], a java-based microarray analysis tool developed by US FDA.

#### 2.2.3. Gene Expression Data Analysis

Global scaling normalization was performed with Median Scaling Normalization in ArrayTrack 3.5.0 using a target median value of 1000. Genes in RGD associated with CVD were selected for further analysis since we focused on the effect of XST on antirat cardiac ischemic injury. Reverse rate (RR) and fold change (FC) were applied to select the differentially expressed genes (DEGs) using an RR > 0.5, and an FC threshold of 1.1. RR was calculated with (1) to evaluate the effect of XST in reversing the changes of gene expression induced by MI modeling as follows:

(1) $$\begin{array}{c} \text{RR}=\frac{M_{i}-X_{i}}{M_{i}-C_{i}}, \end{array}$$

where *C*_*i*_, *M*_*i*_, and *X*_*i*_ are the average expressions of gene *i* in control group, MI group, and XST treatment group, respectively.

#### 2.2.4. Verification with Literature Evidence

The DEGs were differentially expressed due to the whole formulae of XST, and there is a lack of evidence to explain the multicompound and multitarget action of XST. We verified the DEGs through mining existing literatures manually. We downloaded and read abstracts of all articles related to notoginsenoside R1, ginsenoside Rg1, ginsenoside Rb1, ginsenoside Rd, and ginsenoside Re in PUBMED (as of April 10, 2013). If a selected DEG gene is found directly affected by a certain ingredient in literature, it was considered as a potential target of the ingredient and XST. The frequency and its CVD relevancy were recorded. The detailed information of literatures used in this study is listed in supplemental Table S1 (see Supplementary Material available online at http://dx.doi.org/10.1155/2013/652373).

### 2.3. Experimental Validation

The literature information was collected from various sources with some reported in diseases other than CVD. Thus we are interested in validating these results in CVD with *in vitro* experiments. 

#### 2.3.1. Cell Culture

RAW 264.7 cells were obtained from the Cell Bank of Type Culture Collection of the Chinese Academy of Sciences (Shanghai, China) and cultured at 37°C in 5% CO_2_ in DMEM containing 10% heat-inactivated FBS and penicillin/streptomycin. In all experiments, cells were grown to 80–90% confluence and subjected to no more than 15 cell passages.

#### 2.3.2. Western Blot Analysis

The RAW 264.7 cells were plated in 60 mm culture dishes (2 × 10^6^ cells). Twenty-four hours later, cells were incubated with LPS (200 ng/mL) and 50 *μ*M different ingredients of XST or different concentrations of ginsenoside Rd (1, 10, 25, and 50 *μ*M). In our experiments, all ingredients were dissolved in DMSO as 100 mM stocks. All final cell-culture volumes were 5.0 mL, and the cells were incubated at 37°C for twenty-four hours after addition of stimulus. The cells were lysed on ice for 5 min in RIPA lysate with 1 mM PMSF and protease inhibitor and phosphatase inhibitor. The cell lysate solutions were transferred into 1.5 mL polypropylene tubes and the samples were centrifuged for 10 min at 12000 rpm at 4°C. Cell lysate proteins were quantified with the BCA assay (Beyotime Institute of Biotechnology, Jiangsu, China). Twenty *μ*L protein solutions from each culture were electrophoresed into sodium dodecyl sulfate-polyacrylamide gel electrophoresis (SDS-PAGE), and the separated proteins were transferred onto PVDF membranes by iBlot Western Blotting System (Invitrogen). The *β*-actin content of each sample was determined to ensure equal protein loading. The membrane was blocked with 5% skim milk solution in tris-buffered saline (150 mM NaCl, 10 mM Tris-HCl, pH 7.4) with 0.1% Tween 20 (TBST) buffer for 1.5 hour at room temperature (RT). After blocking, the membrane was incubated with primary antibodies against iNOS (1 : 200 diluted in TBST containing 5% skim milk) and *β*-actin (1 : 1000 diluted in TBST containing 5% skim milk) for 3 hours at RT or overnight at 4°C. The membrane was then washed with TBST and incubated with antirabbit (iNOS) or antimouse (*β*-actin) horseradish peroxidase (HRP)-conjugated immunoglobulin G secondary antibodies (1 : 5000 diluted in TBST containing 5% skim milk) for 1.5 hours at RT. The specific proteins were detected using a SuperSignal West Femto Maximum Sensitivity Substrate (Thermo Scientific Inc., Bremen, Germany). Digital images were collected using a Bio-Rad Universal Hood II gel documentation system and the quantitation of protein was evaluated with Quantity One software (Bio-Rad).

### 2.4. Network Construction and Network Analysis

The associated targets of XST and the individual ingredients were subjected to Kyoto Encyclopedia of Genes and Genomes (KEGG) pathway [30] enrichment analysis using ArrayTrack v.3.5.0. The pathways closely related to MI or CVD were selected for further analysis. Based on these results, we constructed the compound-target-pathway network and compound-pathway network using Cytoscape version 2.8.2 [31]. If the interaction between a compound and a target was described in more than one CVD-related literature (including one), the connection between the component and target was marked as solid line. Otherwise, it was marked as a dotted line. The thickness of the lines was proportional to the numbers of related literatures. 

## 3. Results and Discussion

571 genes (721 probe sets) in RGD database were found differentially expressed in this study with RR > 0.5 and FC > 1.1 after the treatment of XST. The gene list can be found in supplementary Table S2. These genes were treated as the potential targets of XST on anti-MI associated with CVD. The genes were further filtered with literature lines of evidence. As a result, 70 potential targets were affected by at least one compound as found in the literature (supplementary Table S3). Among them, TNF-*α* and iNOS were influenced by all the five ingredients and associated with anti-inflammatory activity. I*κ*B, eNOS, caspase-3, JNK, IL-4, SOD, IL-1*β*, and COX-2 were affected by four compounds. Eight targets were affected by three compounds and fourteen targets were influenced by two compounds. The remaining thirty eight were affected by only one compound. 

**Figure 2:**
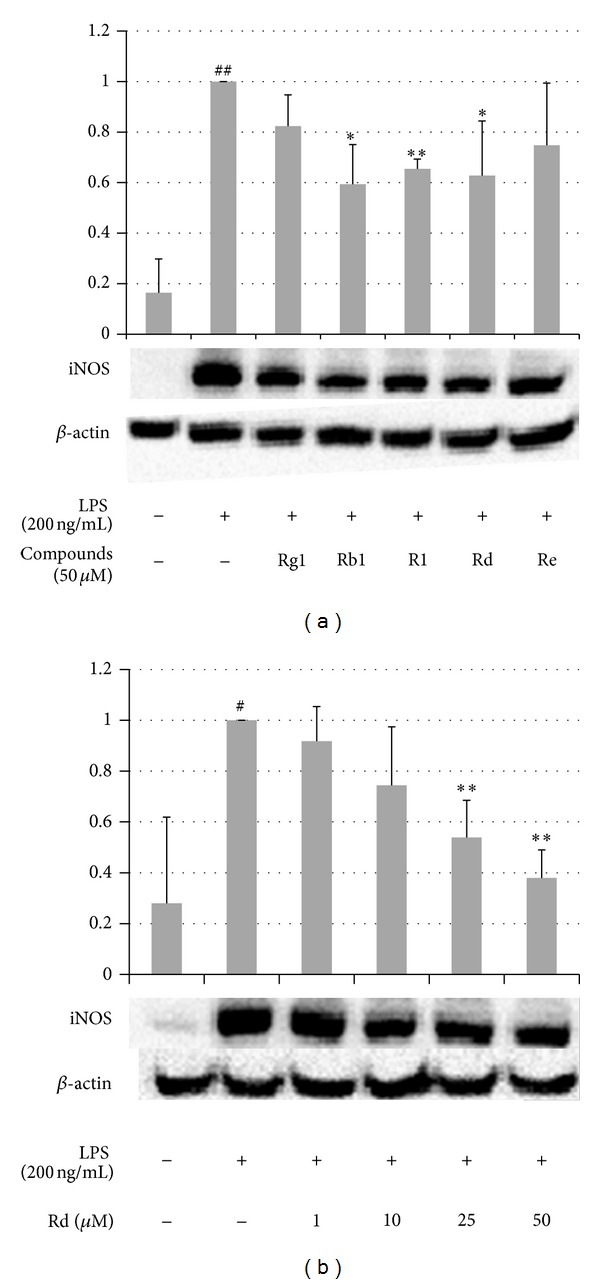
The effect of ingredients of XST on the protein expression of iNOS in LPS-stimulated RAW 264.7 cells. (a) The cells were incubated with LPS (200 ng/mL) and ginsenoside Rg1, ginsenoside Rb1, notoginsenoside R1, ginsenoside Rd, and ginsenoside Re at 50 *μ*M for twenty-four hours. (b) Cells were incubated with LPS (200 ng/mL) and indicated concentrations of ginsenoside Rd for twenty-four hours. The whole cell extracts were prepared, and the expression level of iNOS was determined by Western blot analysis. The values are expressed as the mean ± S.D. from three independent experiments. Statistical significance: *P < 0.05 and **P < 0.01 versus LPS-stimulated cells and ^#^ *P* < 0.05 and ^##^ *P* < 0.01 versus control (nonstimulated cells).

Among the 70 targets of XST, iNOS was affected by all five compounds with four of them reported in noncardiovascular diseases. We selected iNOS as an example to validate its involvement in the mechanism of XST treating MI. LPS-stimulated RAW 264.7 macrophage cell is a commonly used cell model of inflammation, which produces numerous proinflammatory mediators and cytokines and significantly promotes the expression of iNOS protein upon induction. The effects of ginsenoside Rg1, ginsenoside Rb1, notoginsenoside R1, ginsenoside Rd, and ginsenoside Re on iNOS protein expression in RAW 264.7 cells were examined with Western blot analysis. As shown in Figure 2, the expression of iNOS protein was significantly upregulated in response to LPS (200 ng/mL); ginsenoside Rb1 and Rd and notoginsenoside R1 showed significant effects in inhibiting its expression (*P* < 0.05) and ginsenoside Rg1 and Re also showed a decreasing tendency. Furthermore, we studied the dose dependent effect of ginsenoside Rd on iNOS which has not been reported before in CVD-related literatures. We found that ginsenoside Rd dose-dependently downregulated the expression of iNOS as shown in Figure 2. These results indicated a novel compound-target interaction between ginsenoside Rd and iNOS in CVD. Therefore combining lines of evidence from microarray gene expression analysis and the literature survey provided an effective way for finding novel compound-target connections. 

**Figure 3:**
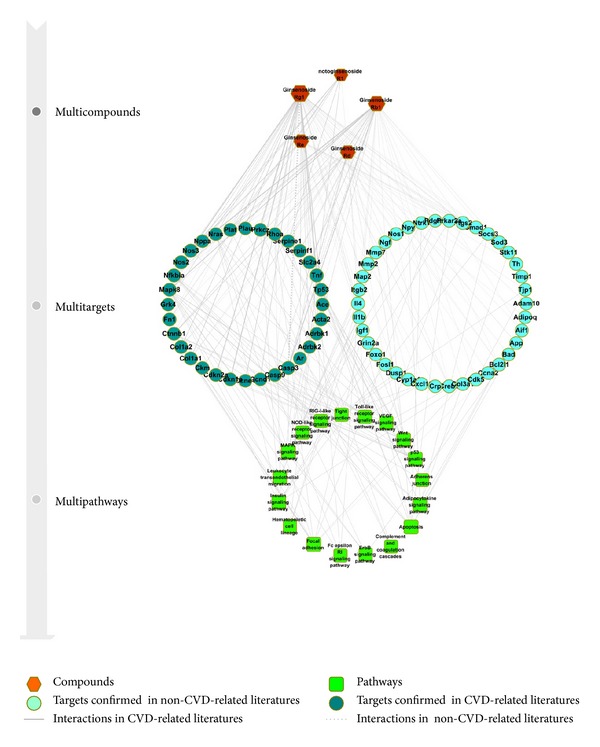
The compound-target-pathway network of XST. The hexagon nodes represent the compounds, the circular nodes represent the targets, and the rounded square nodes represent the pathways. The node size was proportional to the number of interactions between nodes. The line width was proportional to the number of related literatures.

**Figure 4:**
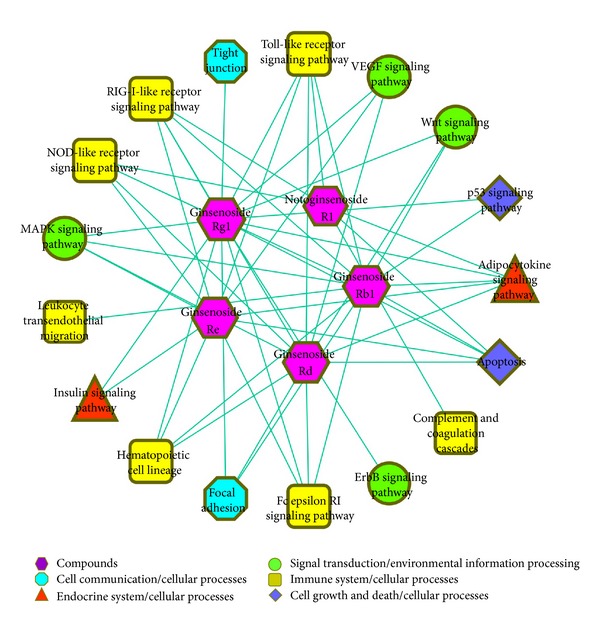
The compound-pathway network of XST. The hexagon nodes represent the compounds. Pathways in different classes were differently colored and shaped. The node size was proportional to the number of interactions between nodes.

Based on the results of gene chip and literatures, we generated the compound-target-pathway network (Figure 3) which consisted of 93 nodes (5 compounds, 70 targets, and 18 pathways) and 240 edges and compound-pathway network (Figure 4) which consisted of 22 nodes (5 compounds and 17 pathways) and 54 edges. Among the eighteen KEGG pathways enriched within all targets of XST, apoptosis and p53 signaling pathways are critical pathways in regulating cell death. It is known in the literature that p53 signaling pathway and apoptosis pathway mediate cardiomyocyte apoptosis and play a decisive role in the progression of pathological remodeling and heart failure following MI [32, 33]. In addition, many inflammatory response related pathways were also enriched, including complement and coagulation cascades, Fc epsilon RI signaling pathway, hematopoietic cell lineage, leukocyte transendothelial migration, NOD-like receptor signaling pathway, RIG-I-like receptor signaling pathway, and Toll-like receptor signaling pathway. It was suggested that XST modulates inflammatory responses in treating ischemia, which has been found to prevent damages of the inflammatory factors to cardiac cells [34–37]. The influencing in complement and coagulation cascades pathway indicated that XST and its constituent compound notoginsenoside R1 are functional in modulating the coagulation process in MI, which is one of the most common clinical risk factors [32–35]. 

Focal adhesion, tight junction, and adherens junction pathways play important roles in maintaining tissue architecture, cell polarity, cell proliferation, and cell death. They may affect the pathologic remodeling of cardiovascular tissues in myocardial infarction [38–40]. Interestingly, adherens junction was not influenced by any component individually but enriched by all the targets in combination. It suggests that XST might modulate adherens junction pathway through a synergistic action, a typical phenomenon of Chinese medicine.

ErbB signaling pathway, MAPK signaling pathway, VEGF signaling pathway, and Wnt signaling pathway may exert pleiotropic effects on cardiovascular cells, regulating cell growth, fibrosis, and inflammation [41–46]. Adipocytokine signaling pathway and Insulin signaling pathway are two pathways involved in energy metabolism such as fatty acid oxidation and glucose uptake and glycogen synthesis, the influence of which could regulate the energy supply in cardiac ischemia. Ginsenoside Rg1 is likely the most important one due to its comprehensive involvement in modulating various targets and pathways.

As shown in Table 1, there were a number of targets influenced by XST and its constituent compounds that were not confirmed in CVD-related literatures. They can serve as targets of priority for experimental validation in the future, and iNOS is such an example validated in this study.

## 4. Conclusion 

In this study, we presented a network-based approach to illustrate the multicompound, multitarget, and multipathway mechanism of Chinese medicine. In a case study of XST, for the first time, we dissect a complex MOA of antimyocardial infarction through multiple targets and pathways with sound experimental lines of evidence. The compound-target-pathway network was constructed to illustrate its multicompound, multitarget, and multipathway regulatory mechanism on anti-MI. Seventy potential targets of XST, of which 32 were experimentally validated either by our *in vitro* assays or by CVD-related literatures, were identified in this study. Specifically, ginsenoside Rb1 and Rd and notoginsenoside R1 were experimentally validated to exert repressive regulatory effects on iNOS in CVD specific context indicating their anti-inflammatory roles. The dose-dependent relation between ginsenoside Rd and iNOS was also validated in this study. Our results indicated that the major ingredients of XST might modulate numerous different targets and pathways involved in inflammation, adhesion, cell proliferation, apoptosis, and energy supply. 

## Supplementary Material

Supplemental Table S1: The detailed information between compounds, targets and literatures used in this study.
Supplemental Table S2: The DEGs list of XST on anti-MI associated with CVD.
Supplemental Table S3: The potential targets of XST validated with literature evidences.

## Figures and Tables

**Table 1:** The number of targets and pathways of XST and the major ingredients. The pathways represent the number of CVD-related pathways enriched by the all targets of XST or the major ingredients.

	Targets			Pathways
	All	Confirmed in CVD-related literatures	Confirmed in non CVD-related literatures	
XST	70	32	38	18
Ginsenoside Rg1	49	17	32	15
Ginsenoside Rb1	36	12	24	12
Notoginsenoside R1	9	8	1	6
Ginsenoside Rd	20	3	17	11
Ginsenoside Re	18	2	16	10
